# Long versus short cephalomedullary nail for trochanteric femur fractures (OTA 31-A1, A2 and A3): a systematic review

**DOI:** 10.1007/s10195-016-0405-z

**Published:** 2016-04-19

**Authors:** John Dunn, Nicholas Kusnezov, Julia Bader, Brian R. Waterman, Justin Orr, Philip J. Belmont

**Affiliations:** Department of Orthopaedic Surgery and Rehabilitation, William Beaumont Army Medical Center, 5005 North Piedras St., El Paso, TX 79920-5001 USA

**Keywords:** Hip fracture, Hospital cost, Cephalomedullary nail, Reoperation

## Abstract

**Background:**

Both long and short cephalomedullary nails (CMN) may be used to treat trochanteric femur fractures. The objective of this paper was to compare the clinical outcomes between long and short CMN in the treatment of trochanteric hip fractures.

**Materials and methods:**

A literature search was performed, identifying 135 papers; 4 of which met inclusion and exclusion criteria. Papers included were those that compared cohorts of long and short nails for stable trochanteric femur fractures of level III evidence or superior. Data was pooled and analyzed, focusing on reoperation rate, secondary femoral shaft fracture rate, estimated blood loss, transfusion rate, operative time and length of stay.

**Results:**

Included in the analysis were 1276 patients, with 438 short and 838 long CMN. The average age was 82.0 years for short CMN and 79.0 years for long CMN (*P* = 0.0002). The average follow up was 18 months, 46 % were male, and 71 % had an ASA (American Society of Anesthesiologists score) classification ≥3. The rate of reoperation was 5.0 % and 3.8 % for short and long CMN, respectively (*P* = 0.31). The rate of refracture was 1.6 % and 0.95 % for short and long CMN, respectively (*P* = 0.41). As compared to long nails, short nails had an average blood loss of 39 mL less (*P* = 0.0003), an 8.8 % decrease in transfusion rate (*P* = 0.07), and incurred 19 min less operative time (*P* < 0.0001). No significant differences between short and long nails were observed for either other complications, hardware complications, non-union, or mortality.

**Conclusions:**

For trochanteric femur fractures, short CMN have a low reoperation rate while significantly decreasing operative time and estimated blood loss with the additional benefit of being cost effective.

**Level of evidence:**

Level 3.

## Introduction

The frequency of hip fractures is increasing steadily with an aging and increasingly physical active population [1, 2]. It is estimated that by 2050, there will be 6.26 million hip fractures world-wide annually. By the age of 80 years, 20 % of women will have sustained a hip fracture, and by 90 years, nearly 50 % of women will have had a hip fracture [3]. Furthermore, the 1-year mortality for hip fractures is roughly 20 % [4, 5].

Stable trochanteric femur fractures are most often fixed by cephalomedullary nails (CMN) or sliding hip screws (SHS). For stable fracture patterns, CMN has been shown to be equivalent to SHS in terms of stability [6]. However, the SHS construct has been found to provide inadequate fixation in more unstable fractures types [7, 8], more often leading to malreduction [9] and lag screw cut-out [10]. CMN have been increasingly favored as a more reliable option for hip fracture fixation, and the utilization of CMN is increasing [11], especially among younger surgeons [12].

Both short and long CMN are available options for hip fracture fixation. Short nails offer the advantages of shorter operative times, reduced blood loss, and lower transfusion rates [13, 14]. Conversely, long nails offer the theoretical benefit of protecting the full length of the femur, particularly in elderly patients with osteoporotic or osteopenic bone, thus potentially decreasing secondary femoral shaft refracture rates [15]. However, because of the limited power of the individual retrospective comparative studies, differences in rates of secondary femoral shaft refracture reoperation have not been found to be significant [13, 14, 16, 17].

We hypothesize that by pooling data from all available comparative cohorts regarding CMN fixation of extra-capsular AO type 31A fractures, there will be no difference in reoperation and secondary femoral shaft refracture rates of short and long CMN. Furthermore, by conducting a number needed to treat to harm and concomitant cost analysis comparing the combined reoperation and secondary femoral shaft refracture rates of the short and long CMN derived from our systematic review, we hypothesize that short CMN are significantly more cost effective.

## Materials and methods

The present study is reported following PRISMA guidelines [18]. There was no source of funding or support for this analysis.

### Eligibility

The inclusion criteria for the present analysis comprised papers that (1) reviewed results of treatment of patients with simple or multifragmentary intertrochanteric femur fractures (31-A1, A2, and A3); (2) compared results from patients treated with long CMN versus short CMN fixation; (3) followed patients for a minimum of 1 year; and (4) included description and rate of reoperation and periprosthetic fracture. Only studies written in the English language were considered.

Studies were excluded if they (1) did not include both short and long CMN cohorts; or (2) had less than 1 year follow up. One paper [16] included fractures other than the 31A type. In this analysis, all non-31A fractures (113 fractures from the study by Vaughn et al. [16]) and the respective data were excluded from the present analysis.

### Outcomes

The primary outcome measures were the rates of (1) reoperation, (2) periprosthetic fracture, and (3) mortality. We additionally isolated reoperation due to nonseptic failure as well as reoperation due to mechanical failures alone. Nonseptic failures excluded infection as a cause for revision. Mechanical failures included failures of the implant, such as screw cut-out, loosening, fixation failure, and prominent screws, excluding revision for pain without implant failure. The secondary outcome measures were (1) blood loss, (2) number of RBC units transfused, (3) number of patients transfused, (4) operative time, (5) length of hospital stay, (6) nonunion, and (7) complications. We isolated complications other than periprosthetic fracture as well as hardware complications specifically. Demographic data were also collected and pooled.

### Search strategy and selection of studies

A systematic search was performed for all articles published on the treatment of intertrochanteric femur fractures with cephalomedullary fixation using the PubMed, Medline, EMBASE, and Cochrane databases between the years 1990 and 2015. Search terms included, cephallomedullary*, intertroch*, trochanteric fracture nail*, extracapsular fracture*, short*, and long*.

The abstracts generated by the search were individually assessed for relevance by two senior authors (B.R.W. and P.J.B.). Full manuscripts of individual studies were then thoroughly reviewed independently according to the inclusion and exclusion criteria. If the data was not explicitly stated in the manuscript, the corresponding author was contacted for further information. Any disagreements or discrepancies in study selection were moderated by consensus.

### Assessment of methodological quality and data collection

The GRADE (Grading of Recommendations Assessment, Development and Evaluation Working Group) criteria are a quality assessment template used to evaluate the quality of methods in study analysis [19]. Using this template, the quality of the selected studies was independently assessed by the two senior authors (B.R.W. and P.J.B.). Disagreement concerning study quality was moderated by consensus. For all previously identified studies deemed eligible, the authors extracted pertinent data.

### Data pooling across studies and data analysis

Demographic data, primary outcome measures, and secondary outcome measures from comparable studies were pooled for all patients, those receiving a short CMN, and those receiving a long CMN. None of the studies received external funding and no clear sources of bias were identified. Outcome measures were compiled and compared.

### Statistical analysis

Continuous variables were compared with use of the Student* t* test, and categorical data were compared with use of either the Fisher exact test or the Chi square test. A *P* value of < 0.05 was considered significant. Analyses were performed using SAS version 9.3 (SAS Institute, Cary, NC).

A number needed to harm analysis was conducted given the absolute risk reduction in both refracture and reoperation between the short and long CMN cohorts. Furthermore, we performed a post hoc power analysis given the existing data to determine the sample size necessary to produce statistical significance between the short and long CMN cohorts with respect to both refracture and reoperation.

## Results

**Fig. 1:**
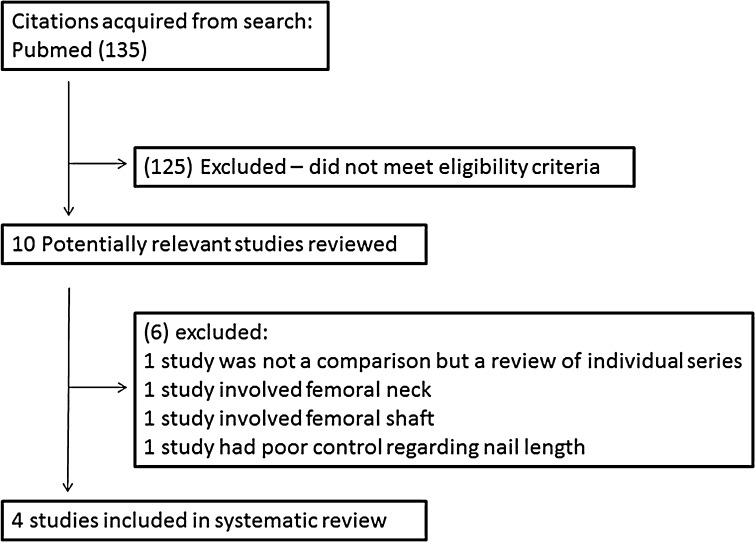
Cohort inclusion and exclusion

The search resulted in 135 potentially eligible studies, while only 4 met inclusion and exclusion criteria (Fig. 1). All studies were retrospective comparative cohorts, level III therapeutic studies [13, 14, 16, 17] (Table 1). In total, 1179 patients, with 438 short and 838 long CMN, were included. Patients were treated with one of the following four CMN: Gamma 3 short (Stryker, Kalamazoo, MI), gamma 2 and 3 long nails (Stryker), or Synthes Trocanteric Fixation Nail short or long nail (Synthes, Paoli, PA). The average age was 82.0 years and 79.0 years, for short and long nails, respectively (*P* = 0.0002). The average follow-up was 18 months, 46 % were male, and 71 % had an ASA (American Society of Anesthesiologists score) classification ≥3 (Table 1).

**Table 1:** Demographics. *ASA* American Society of Anesthesiologists score,*NR* not reported

Author	Fractures	Follow-up (months)	Male	Age (years)	ASA ≥3	OTA classification (31A1/A2/A3)
Hou et al. [14]	283	37	73	79	171	126/157/0
	100 short			81		59/41/0
	183 long			78.6		67/116/0
Boone et al. [13]	194	12	54	81.1	NR	59/142/0
	82 short			83.3		31/51/0
	119 long			79.6		28/91/0
Vaughn et al. [16]	143	12	NR	NR	NR	36/79/28
	37 short					11/19/7
	106 long					25/60/21
Kleweno et al. [17]	559	12	349	84	426	NR^a^/NR^a^/143
	219 short					NR
	430 long					NR

^a^Reported 416 31A1/A2 fractures, combined

### Outcome measure reporting

Intraoperative variables and hospital length of stay were recorded (Table 2). Two studies reported estimated blood loss, transfusion rates, and length of stay [13, 14]. The average blood loss was 86.7 and 135.2 mL for short and long CMN, respectively (*P* = 0.0003). The blood transfusion rate was 41 % for short and 50 % for long CMN (*P* = 0.07). The length of hospital stay was 7.0 and 7.3 days for short and long CMN, respectively (*P* = 0.48). Three studies reported operative time [13, 14, 17]. The mean operative time was 47.1 min for short CMN and 65.6 min for long CMN (*P* < 0.0001).

**Table 2:** Intraoperative variables and hospital length of stay

	Short nail	Long nail	*P* value
Age, mean (SD)	82.0 (8.1)	79.0 (9.1)	0.0002
EBL (mL), mean (SD)	96.7 (67.2)	135.2 (139.7)	0.0003
RBC (units)			0.8166
1–2	37 (37.0 %)	68 (37.2 %)	
3–5	4 (4.0 %)	10 (5.5 %)	
>5	1 (1.0 %)	5 (2.7 %)	
Patients transfused	75 (41.2 %)	151 (50.0 %)	0.0738
Operative time (min), mean (SD)	47.1 (18.4)	65.6 (32.5)	<0.0001
Length of stay (days), mean (SD)	7.0 (4.5)	7.3 (4.1)	0.4737

All studies recorded reoperation, refracture, and other complications (Table 3). The overall rate of reoperation was 5.0 and 3.8 % for short and long CMN, respectively (*P* = 0.31). The rate of reoperation due to non-septic failure was 4.8 % for short and 3.3 % for long CMN (*P* = 0.20) while that due to secondary to mechanical failure was 3.7 % and 2.5 % for short and long CMN, respectively (*P* = 0.25).

**Table 3:** Refracture, reoperation, and complication rates

	Short nail	Long nail	*P* value
Reoperation	22 (5.02 %)	32 (3.82 %)	0.3103
Reoperation due to aseptic failure	21 (4.79 %)	28 (3.34 %)	0.1996
Reoperation due to mechanical failure	16 (3.65 %)	21 (2.51 %)	0.2463
Refracture	7 (1.60 %)	8 (0.95 %)	0.3112
Other complications	20 (4.57 %)	44 (5.25 %)	0.5949
Hardware complications	14 (3.20 %)	32 (3.82 %)	0.5713
Nonunion	1 (0.23 %)	5 (0.60 %)	0.3611
Mortality	22 (5.02 %)	42 (5.01 %)	0.9932

The rate of refracture was 1.60 % for short CMN and 0.95 % for long CMN (*P* = 0.31). The rate of other complications was 4.6 % and 5.25 % for short and long CMN, respectively (*P* = 0.57). There was no statistically significant difference between short and long CMN for other complications, hardware complications, non-union, or mortality.

Although the refracture and reoperation were not statistically significant between the two groups, a number needed to harm was calculated as a worst case scenario. The calculated number needed to treat to harm for refracture and all-cause reoperation for short over long CMN were calculated to be 154 and 83, respectively.

The studies did not report mortality uniformly. Vaughn et al. [16]. did not report mortality. In the study by Kleweno et al. [17], the authors found that 175 of 698 patients died prior to 12-month follow up. These patients were excluded from any further analysis. Similarly, Boone et al. [13]. did not distinguish between short and long CMN patient mortality but noted that 41 of 194 patients died within 1year. Conversely, Hou et al. [14]. reported 9/58 short and 15/68 long CMN deaths within 1 year. While the mortality rate between long and short CMN could not be compared in this analysis, the pooled 1-year mortality rate was 26.5 %.

## Discussion

In this systematic review, there was a small but statistically insignificant increase in all-cause reoperation and secondary femoral shaft refracture with short versus long CMN in the setting of stable trochanteric femur fractures. The current study only includes the most third generation of the short CMN, including the Stryker Gamma 3 short and Synthes Trochanteric Fixation Nail short, which have been reported to have decreased rates of post-operative secondary femoral shaft fractures [15]. An argument could be made that the average 38.5 mL increase in estimated blood loss and 18.5 min increase in operative time with implantation of a long versus short CMN is not clinically significant. However, the surgeon must consider the increased cost associated with use of the long CMN.

The higher cost of the long CMN is most heavily influenced by the increased operative time (Table 4). The difference in operative time between the short and long CMN is most likely attributed to additional time spent reaming the canal for the long CMN, and the method of the distal interlock screw insertion. The cost of running the operating room depends on many factors, including type and complexity of surgical procedure, fixed versus variable overhead costs, and the professional fees of the surgeon and anesthesia provider [20]. It is estimated that operating rooms cost, on average, US $62 min^−1^; ranging from as low as US $22 to as high as US $133 min^−1^ [20]. The true cost to the hospital is unpublished as institutions do not typically openly disclose profit margins. These figures do not account for implant costs and provider fees.

**Table 4:** Long cephalomedullary nails (CMN) cost analysis (US $)

Contributing factors	Cost	Calculation	Additional cost for long CMN
Operative time	$62/min	$62/min × (65.6–47.1 min)	$1147
Provider fees			
Orthopaedic surgeon	$207/h	($329/h = $5.5/min) × (65.6–47.1 min)	$101
Anesthesia	$122/h		
Total provider fee	$329/h		
Implant cost			
Long CMN	$2400	$(2400 − 1800) + $230 + $130	$960
Short CMN	$1800		
Additional locking screw	$230		
Reaming Rod	$130		
Total cost	–	$1147 + $101 + $960	$2208 (per long CMN)
Overall cost per reoperation	–	$2208 × 83	$183,264
Overall cost per refracture	–	$2208 × 154	$340,032
Average cost of reoperation	$30,000	–	–
Difference in cost for reoperation	–	$183,264/$30,000	6.1-fold
Difference in cost for refracture	–	$340,032/$30,000	11-fold

Provider fees vary based on means of compensation from fixed salary to hourly wages. In a review of two anesthesia departments in academic institutions, the hourly staffing cost was found to range from US $111 to $176 with a median of $122 [21]. Though the pay per minute for orthopaedic surgeons in the operating room is unpublished, the median hourly pay of an orthopaedic surgeon in the United States is $204–210 [22, 23]. Therefore, provider fees would foreseeably cost another ($122 + $204)/60 min = $5 per minute to the hospital. Combined with the cost of running the operating room for an additional 18.5 min, the long CMN would cost an additional ($62 + $5) × 18.5 min = $1248. These estimates are still conservative as they do not include costs for other personnel including operating room and recovery nursing, surgical technicians, other medical staff who may be required to treat the effects of longer surgeries with more blood loss, and the associated costs of increased blood transfusions.

Finally, with regard to the differences in implant pricing, at our institution the average long CMN costs roughly $2400 while the short CMN costs $1800 (Depuy Synthes; https://www.depuysynthes.com/). The cost of a long nail is further increased by the additional locking screw ($230) and reaming rod ($130). Altogether, considering the basic pricing differences, locking screw, and reaming rod, a long CMN costs approximately $960 more than a short nail. Combining the aforementioned factors again yields a conservative cost estimate of ($1248 + $960) = $2208 more for utilization of a long CMN compared to a short CMN.

Taking a number needed to treat to harm for refracture of 154, assuming the refracture requires reoperation, the total additional cost is ($2208 × 154 =) $340,032. Taking the lower NNTH for all-cause reoperation (83), by the same calculation we find an additional cost of $183,264 for the long CMN. Therefore, whether considering reoperation in general or reoperation only after refracture, the additional cost of long CMN is considerable.

This must be weighed against the cost of revision of a periprosthetic fracture following use of a CMN. Revision of a periprosthetic fracture is costly and not without complication. However, the cost of revision surgery (refixation or arthroplasty) for failure of primary fixation in 2014 was found to be on average $30,000 (revision hip arthroplasty ranging from $20,000 to $40,000) [24–29]. This number pales into comparison to the added overall cost of the long CMN, and this is based on a conservative estimate. In addition, the charge to the patient could easily be up to five- or six-fold this amount. Therefore, from a cost-benefit analysis standpoint, the cost of using long CMN over 154 cases represents roughly five- to ten-times the cost of using short nails over the same period and having one revision.

There are several other factors that favor short nails. First, short nails are technically easier as the inter-lock screws may be placed with the help of a jig. This allows lower-volume surgeons to safely and efficiently lock the nail distally. Second, short nails have demonstrably less blood loss and need for transfusion [13, 14]. These factors make short nails especially more attractive in the older patient with multiple medical comorbidities. The cumulative effect of these factors is not known. In addition, given the findings of the power analysis, which demonstrated that nearly 8500 patients would be needed to reach true statistical significance, it is likely that the statistically insignificant differences in refracture and reoperation rates are not clinically significant to many orthopaedic surgeons.

The primary limitation of this systematic review is the limited power. A large multi-center database study would be necessary to prove statistical significance for refracture and reoperation rates. Given the increased blood loss, operative time and fiscal cost associated with long compared to short CMN, regardless of whether refracture or reoperation rates are proven significantly higher by a better powered analysis, there is still a role for the short CMN as a faster, safer, less expensive, and less invasive option for patients with an trochanteric femur fracture without subtrochanteric extension.
